# 

**Published:** 1893-10-07

**Authors:** 


CONTENTS.

Mr. T. Holmes on John Hunter - 1

Around the Hospitals - 2

The Microscope in Relation to Modern Biology - 3

Famous Poisoners in Fiction. VII.—The Child of Crime - 4

Modern Aspects of Diabetes - 5

The Field of Science - 6

Annotations.—The late Professor Jowett—Huxley and Clericalism—Dr. Bristowe and his Friends - 7

The Hospital Clinic—Ingrowing Toe-nail.—II. Treatment - 9

The Paddington Green Children's Hospital.—The Treatment of Nævi - 10

New Drugs and Preparations.—The Use and Administration of Creasote - 11

Notes and News - 8

Editor's Letter-box.—Caries of the Spine - 12

The London and Counties Medical Protection Society. 12

The Institutional Workshop—

Hospital Finance.—Cost of Administration.—Hospitals for Consumption - 13

Practical Departments.—Poultice Warmer and Inhaler—New Bed Rest - 14

St. Thomas's Hospital.—Presentation to Dr. Bristowe - 15

Hospital Administration.—Construction Notes - 16

Scraps and Gleanings - 16

The Book World of Medicine and Science - vii

Medical Vacancies and Appointments - ix

EXTRA SUPPLEMENT.—THE NURSING MIRROR.

News from the Nursing World.—The Nurse Dolis—Delicate Children—What Becomes of Old Linen?—Nurses' Co-operation—Rural Requirements—Workhouse Nurses and Certificates—Medical Women—Another Queen's Nurse—Nursing at Washington—Qualified for India—Private Nurses in India—Inaccuracies—Short Items - i

On the Nursing of Diseases of the Nervous System.—I. Introductory - iii

Nursing in Paris.—III. A Visit to the Children - iv

An Isolated Small-pox Case - iv

Appointments - v

Nursing in Ceylon.—The Ceylon Nurses' Association - v

Nursing in South Africa.—Private Nursing - v

Irish Hospitals.—A Visit to a Cork Hospital - vi

Everybody's Opinion.—Deterioration of Nurses—Every-day Dangers—The World of Nurses - vi

Minor Appointments - vii

For Reading to the Sick.—Clouds - vii

Notes and Queries - vii

The Muses' Looking Glass.—"Dodo—A Detail of the Day" viii

Wants and Workers - viii

The Book World for Women and Nurses - ix

